# Inferring linkage disequilibrium from non-random samples^†^

**DOI:** 10.1186/1471-2164-11-328

**Published:** 2010-05-26

**Authors:** Minghui Wang, Tianye Jia, Ning Jiang, Lin Wang, Xiaohua Hu, Zewei Luo

**Affiliations:** 1 School of Biosciences, The University of Birmingham, Edgbaston, Birmingham, B15 2TT, UK; 2 Laboratory of Population & Quantitative Genetics, Institute of Biostatistics, School of Life Sciences, Fudan University, Shanghai 200433, China

## Abstract

**Background:**

Linkage disequilibrium (LD) plays a fundamental role in population genetics and in the current surge of studies to screen for subtle genetic variants affecting complex traits. Methods widely implemented in LD analyses require samples to be randomly collected, which, however, are usually ignored and thus raise the general question to the LD community of how the non-random sampling affects statistical inference of genetic association. Here we propose a new approach for inferring LD using a sample un-randomly collected from the population of interest.

**Results:**

Simulation study was conducted to mimic generation of samples with various degrees of non-randomness from the simulated populations of interest. The method developed in the paper outperformed its rivals in adequately estimating the disequilibrium parameters in such sampling schemes. In analyzing a 'case and control' sample with *β*-thalassemia, the current method presented robustness to non-random sampling in contrast to two commonly used methods.

**Conclusions:**

Through an intensive simulation study and analysis of a real dataset, we demonstrate the robustness of the proposed method to non-randomness in sampling schemes and the significant improvement of the method to provide accurate estimates of the disequilibrium parameter. This method provides a route to improve statistical reliability in association studies.

## Background

Linkage disequilibrium (LD) has long been one of the central topics in evolutionary and population genetics. Linkage disequilibrium refers to non-random association of alleles at different linked or unlinked loci in a population. Inference about LD provides useful information for distinguishing between alternative evolutionary models of genetic polymorphisms within or divergence between populations [1]. The current surge of population based association studies has reported identification of causal genetic variants of disease susceptibilities in humans [2] and complex genetic variation in plants and animals [3,4]. The kernel of these studies is inference of LD between the genetic variants and functional loci that are closely genetically linked. Thus, adequate prediction of LD is obviously crucial for reliability and accuracy of these studies.

The coefficient of LD between two biallelic loci is defined as *D *= *f*_*AB *_- *f*_*A *_*f*_*B *_in a randomly mating population, where *f*_*AB*_, *f*_*A *_and *f*_*B *_are frequencies of gametes *AB*, alleles *A *and *B *in the population. The genetic parameter has been re-parameterized into different forms for various purposes of LD analysis [5]. Hill proposed the well known "chromosome counting" method to estimate the parameter by using data of genotypes at the two loci from a random sample [6]. It has been widely used in population genetic analyses and studies on linkage disequilibrium based mapping [7,8]. The principle of the analysis has also been employed to develop widely used methods for predicting haplotypes of DNA markers in natural populations [9,10]. In practice, however, it is very rare that the samples for LD analyses are truly randomly collected from the population under study. For example, the samples used in many association studies or population genomics analyses were so collected that the frequencies of some genotypes are artificially inflated to ensure that genotypes involving a rare allele are well represented [11,12]. Weir and Cockerham explored the consequences of implementing the method to estimate LD by using the samples in which some genotypes are missing and stressed that the method should not be used to estimate the parameter from non-random samples [13], but neither appropriate theory nor method has been developed for inferring population disequilibrium from non-random samples.

In this paper, we develop a new method to calculate the maximum likelihood estimate of the coefficient of linkage disequilibrium between genes at any pair of polymorphic loci in any randomly matting population by making use of samples of genotype data, which are non-randomly collected from the population of interest. On the basis of simulation studies, we demonstrate that bias in estimates of the disequilibrium parameter from Hill's method arising from the use of non-random samples can be substantially reduced by implementing the new method. We compared analyses of a 'case and control' dataset of *β-*thalassemia using three different methods: Hill's, haplotype prediction by the computer software PHASE2.1.1 and the method developed in the present study.

## Results

We developed a likelihood-based statistical approach to estimate the coefficient of linkage disequilibrium between a pair of polymorphic loci in a natural population and to test for significance of the disequilibrium by making use of the samples that are not randomly collected from the population. The method uses information from the conditional distribution of genotypes at one locus given genotypes at the other in formulating the statistical analysis with non-random samples. This is in contrast to the approach proposed by Hill [6], which relies on information of a joint distribution of genotypes at the polymorphic loci and estimates the disequilibrium parameter from using the samples randomly collected from the population under question. Hill's method has been extended or converted into various forms/approaches that are widely used in the current surge of genetic association studies and population genetic analyses [11,12,14-18]. Here, our analysis is focused on Hill's method (*H*) and the method (*L*) developed in the present study.

To explore the adequacy of the methods *H *and *L *in estimating LD and their statistical power in detecting LD, we first conducted a simulation study to generate samples collected from the simulated population by sampling with various degrees of non-randomness. We then implemented the methods to analyze both the simulated datasets and the real data of 20.693 kb DNA sequence surrounding the *β*-globin gene from a 'case and control' study with *β*-thalassemia [19].

### Simulation Study

The study considered three schemes of sampling individuals from a simulated population in which the distribution of genotypes at the marker and disease loci was as described by Table 1 for any given set of simulation parameters *p*, *q *and *D*. Sampling Scheme I involved *n *individuals being sampled completely randomly from the simulated populations. Sampling Scheme II generated the samples in which either individuals with a specific marker genotype or individuals with a specific marker-disease genotype were missing. Finally, Sampling Scheme III mimicked the generation of the case-control samples used in most association studies, in which the cases and controls were present in the sample in equal proportions. In addition, we explored influence of various 'case and control' proportions on statistical power of the 'case and control' design for detecting LD. The computer programs designed for simulating linkage disequilibrium between two bi-allelic loci can be found elsewhere [20], and were modified to generate the samples in the present study.

**Table 1:** Conditional probability distribution of disease genotypes for a given marker genotype

***MM***			***Mm***			***mm***		
***AA***	***Aa***	***aa***	***AA***	***Aa***	***aa***	***AA***	***Aa***	***aa***

*Q*^2^	2*Q*(1 - *Q*)	(1 - *Q*)^2^	*QR*	*Q *+ *R *- 2*QR*	(1 - *Q*)(1 - *R*)	*R*^2^	2*R*(1 - *R*)	(1 - *R*)^2^
*n*_11_	*n*_12_	*n*_13_	*n*_21_	*n*_22_	*n*_23_	*n*_31_	*n*_32_	*n*_33_

*Q *= *q *+ *D*/*p *and *R *= *q *- *D*/(1 - *p*)

Table 2 lists the means and standard deviations of 1,000 estimates of the linkage disequilibrium coefficient from Hill's method (Method *H*) and the method proposed in the present study (Method *L*) by making use of random samples of 200 individuals from 12 different simulated populations (Sampling Scheme I). Allele frequencies at the marker and disease loci were directly calculated from the samples. Both methods adequately estimate the disequilibrium parameters although the estimates from Method L may have slightly smaller standard deviations than those from Method *H*.

**Table 2:** Prediction of Sampling Scheme I

Pop.	*p*	*q*	(*D*_*min*_, *D*_*max*_)	*D*	± *s.d.*	± *s.d.*
1	0.5	0.5	(-0.25, 0.25)	0.20	0.1999 ± 0.0078	0.2004 ± 0.0078
2	0.5	0.5	(-0.25, 0.25)	0.10	0.1002 ± 0.0145	0.1003 ± 0.0145
3	0.3	0.3	(-0.09, 0.21)	0.09	0.0898 ± 0.0133	0.0899 ± 0.0125
4	0.7	0.7	(-0.09, 0.21)	0.09	0.0895 ± 50.0133	0.0896 ± 0.0126
5	0.3	0.5	(-0.15 0.15)	0.10	0.0997 ± 0.0120	0.0998 ± 0.0111
6	0.5	0.3	(-0.15, 0.15)	0.10	0.0995 ± 0.0121	0.0993 ± 0.0109
7	0.5	0.5	(-0.25, 0.25)	-0.20	-0.1995 ± 0.0081	-0.1998 ± 0.0081
8	0.5	0.5	(-0.25, 0.25)	-0.10	-0.0996 ± 0.0146	-0.0997 ± 0.01460
9	0.3	0.3	(-0.09, 0.21)	-0.09	-0.0896 ± 0.0074	-0.0899 ± 0.0068
10	0.7	0.7	(-0.09, 0.21)	-0.09	-0.0897 ± 0.0073	-0.0899 ± 0.0065
11	0.3	0.5	(-0.15 0.15)	-0.10	-0.1000 ± 0.0124	-0.1000 ± 0.0117
12	0.5	0.3	(-0.15, 0.15)	-0.10	-0.0995 ± 0.0120	-0.0993 ± 0.0111

Linkage disequilibrium parameters were estimated based on 1000 simulations of *n *= 200 individuals from 12 different populations: *p *and *q *are the frequencies of alleles of the marker (*M*) and trait (*A*), *D *is the coefficient of linkage disequilibrium between the marker and the disease loci, *D*_*min *_and *D*_*max *_are respectively the minimum and maximum possible coefficients of linkage disequilibrium given allelic frequencies *p *and *q*,  and  are the estimates from Methods H and L respectively, and the means and standard deviations, *s.d.*, calculated from 1000 simulations.

In Sampling scheme II, individuals were randomly sampled from 6 simulated populations but those with either a specific marker genotype (*n*_*i• *_= 0, *i *= 1, 2, 3 corresponding to *MM*, *Mm *or *mm*) or a marker-disease genotype (*n*_*ii *_= 0, *i *= 1, 2, 3 corresponding to *MMAA*, *MmAa *or *mmaa*) were excluded in the sampling process. The sampling continued until the required number of individuals were obtained (*n *= 200). From the samples so obtained, we calculated the allele frequencies, *p *and *q*, and then estimated *D *by use of both methods (*H *and *L*). Means and standard deviations of the *D *estimates, based on 1,000 simulations, are summarized in Table 3. In most cases, the two methods accurately estimate the disequilibrium parameters. However, when individuals with heterozygous genotypes at the marker locus were absent (*n*_2• _= 0) in the sample, Method *H *underestimated the parameters in the simulated populations 3-5. In contrast, Method *L *estimated the parameters adequately in these cases. In the cases where individuals with a specific marker-disease genotype were not present in the sample, Method *H *severely underestimated the disequilibrium, for instance, when *n*_11 _= 0 in simulated population 4 and *n*_33 _= 0 in simulated population 3. The biased estimates were substantially improved by making use of Method *L*. One may argue that different performance of the two methods could be due to the limited sample size (*n *= 200). We compared the methods with using much larger sample sizes (*n *= 400, 800) and observed that the same pattern in mean estimates of the disequilibrium parameter as demonstrated in Table 3 with *n *= 200 (Additional file 1). Another question that may naturally rise from this sampling scheme is how much genotypic distribution at the simulated marker and disease loci deviates from that expected under Hardy-Weinberg equilibrium. The significance test shows that these genotype distributions in the samples generated from the sampling schemes highly significantly deviate from the Hardy-Weinberg expectations (Additional file 2), revealing remarkable non-randomness of the samples with respect to the populations from which these samples were collected.

**Table 3:** Prediction of Sampling Scheme II

Pop.	*D*		*n*_1• _= 0	*n*_2• _= 0	*n*_3• _= 0	*n*_11 _= 0	*n*_22 _= 0	*n*_33 _= 0
1	0.20	± *s.d.* ± *s.d.*	0.18 ± 0.01 0.20 ± 0.03	0.20 ± 0.01 0.20 ± 0.01	0.18 ± 0.01 0.20 ± 0.03	0.17 ± 0.01 0.18 ± 0.04	0.17 ± 0.01 0.17 ± 0.01	0.17 ± 0.01 0.18 ± 0.04
2	0.10	± *s.d.* ± *s.d.*	0.09 ± 0.02 0.10 ± 0.02	0.10 ± 0.01 0.10 ± 0.01	0.09 ± 0.02 0.10 ± 0.02	0.05 ± 0.02 0.06 ± 0.02	0.07 ± 0.01 0.07 ± 0.01	0.05 ± 0.02 0.06 ± 0.02
3	0.09	± *s.d.* ± *s.d.*	0.08 ± 0.02 0.09 ± 0.01	0.06 ± 0.01 0.10 ± 0.04	0.10 ± 0.02 0.09 ± 0.01	0.07 ± 0.01 0.08 ± 0.01	0.04 ± 0.01 0.09 ± 0.06	0.00 ± 0.02 0.04 ± 0.01
4	0.09	± *s.d.* ± *s.d.*	0.10 ± 0.02 0.09 ± 0.01	0.06 ± 0.01 0.09 ± 0.01	0.08 ± 0.01 0.09 ± 0.01	0.00 ± 0.02 0.04 ± 0.02	0.04 ± 0.01 0.06 ± 0.01	0.07 ± 0.01 0.08 ± 0.01
5	0.10	± *s.d.* ± *s.d.*	0.08 ± 0.01 0.10 ± 0.01	0.06 ± 0.01 0.10 ± 0.01	0.12 ± 0.01 0.10 ± 0.01	0.07 ± 0.01 0.08 ± 0.01	0.07 ± 0.01 0.08 ± 0.01	0.05 ± 0.02 0.06 ± 0.02
6	0.10	± *s.d.* ± *s.d.*	0.09 ± 0.01 0.10 ± 0.01	0.10 ± 0.01 0.10 ± 0.01	0.09 ± 0.01 0.10 ± 0.01	0.07 ± 0.01 0.08 ± 0.01	0.07 ± 0.01 0.08 ± 0.01	0.05 ± 0.02 0.06 ± 0.02

Means and standard deviations of coefficients of linkage disequilibrium were estimated from 1,000 repeats for Methods *H *and *L *when 200 individuals were generated from the Sampling Scheme II in which either a marker genotype (*n*_i• _= 0, *i *= 1,2,3) or a marker-disease genotype (*n*_ii _= 0, *i *= 1,2,3) was missing. The population numbers are the same as those in Table 2 and *D *is the true value of the disequilibrium coefficient.

Sampling scheme III considered the scenario that the disease allele had a low frequency but generated an equal number of the case individuals (i.e. either a homozygote or a heterozygote of the disease allele) and the controls (i.e. a homozygote of the wild type allele). To mimic the sampling scheme, we randomly generated a given number of 'case' or 'control' individuals for each of seven simulated populations. From these individuals, 100 'case' and another 100 'control' individuals were randomly collected, making a constant sample size of 200. The samples so generated make a severely non-random collection of the cases but they present a typical example of the samples widely used in many current genetic association studies with a case-control design in which roughly an equal number of sporadic case individuals and control individuals were collected from the population under question [11,14-19]. Use of the samples so collected raised a question of how the allele frequency parameters, *p *and *q*, can be calculated and used to estimate the disequilibrium parameter, *D*. We proposed to calculate *p*, the marker allele frequency, from the control sub-samples, and explored two alternative ways to obtain the value of *q*, the disease allele frequency. Firstly, *q *was obtained from an independent population survey such as a prior epidemiological study or population survey. To assimilate this scenario, we used the stimulated value of *q *in estimation of *D*. Secondly, we explored the use of *q *values directly estimated from the case-control sample in estimation of *D*.

Table 4 compares the means and standard deviations of the LD estimates, *D*, using Methods *H *and *L *on the data from the 1,000 case-control simulations. Although the disease allele frequencies (*q*) were obtained from true value of simulated parameter (i.e. from a population survey in practice), estimates of the disequilibrium coefficient using Method *H *() were seriously biased from their corresponding true values, sometimes even outside the theoretical boundaries for the parameters. In contrast, the Method *L *estimated the disequilibrium parameters (*D*_*L*_) adequately in all seven simulated situations. When the disease allele frequencies were estimated directly from the case-control samples and incorporated into the algorithms to estimate the parameter *D*, both the methods yielded biased estimates of the simulated parameters. However, the estimates from Method *L *deviate less from the corresponding true values than those from Method *H*. It should be noted that Method *H *produced biased estimates from the case-control samples and the bias was more severe when the population frequency of the disease allele was used than when the frequency was directly estimated from the case-control samples. An detailed examination found that this was because, in the former, Method *H *always produced the estimates of the haplotype frequency, , which were outside their theoretical boundaries.

**Table 4:** Prediction of Sampling Scheme III

*p*	*q*	(*D*_*min*_, *D*_*max*_)	*D*	*q *was from population survey		*q *was from sample estimation	
				
				± *s.d.*	± *s.d.*	± *s.d.*	± *s.d.*
0.6	0.005	(-0.003,0.002)	-0.002	-0.011 ± 0.149	-0.002 ± 0.000	-0.113 ± 0.015	-0.071 ± 0.011
0.5	0.01	(-0.005,0.005)	0.004	0.280 ± 0.011	0.004 ± 0.001	0.108 ± 0.013	0.080 ± 0.013
0.5	0.02	(-0.010, 0.010)	0.008	0.273 ± 0.010	0.008 ± 0.001	0.110 ± 0.014	0.081 ± 0.013
0.3	0.03	(-0.009, 0.021)	0.010	0.191 ± 0.026	0.011 ± 0.002	0.104 ± 0.019	0.057 ± 0.018
0.7	0.04	(-0.028, 0.012)	0.010	0.309 ± 0.016	0.011 ± 0.002	0.071 ± 0.012	0.061 ± 0.017
0.3	0.05	(-0.015, 0.035)	0.020	0.192 ± 0.044	0.021 ± 0.004	0.122 ± 0.017	0.066 ± 0.017
0.5	0.10	(-0.050, 0.050)	0.040	0.227 ± 0.008	0.045 ± 0.006	0.124 ± 0.014	0.088 ± 0.012

Simulation parameters for the case-control sampling scheme and means and standard deviation of estimates of the disequilibrium coefficients,  and , from Methods *H *and *L *respectively. *p*, *q *and *D *are simulated values of population allele frequencies at the marker and disease loci and linkage disequilibrium respectively. (*D*_*min*_, *D*_*max*_) are the theoretical minimum and maximum values of *D*. The disequilibrium coefficients were calculated when the marker allele frequency, *p *was calculated directly from the control sub-samples while the disease allele frequency *q *was either from population survey or directly estimated from the case-control samples.

To explore influence of population size of the case-control samples on estimation and detection of the disequilibrium, we calculated estimates of the disequilibrium coefficient and the corresponding LOD scores using Method *L *in the case-control samples with an equal proportion of 'case and control' individuals but different total sample sizes. Table 5 summarizes the means and standard deviations of 1,000 repeated estimates of the disequilibrium coefficients and the corresponding LOD scores. It can be seen that the disequilibrium parameters are estimated adequately from the method by use of the case-control sample with a size as small as only 100 individuals. The LOD scores and the test statistic for significance of the disequilibrium, increase as the sample size increases.

**Table 5:** Prediction from case-control samples with various sample sizes

*p*	*q*	*D*	*n *= 100	*n *= 200	*n *= 400	*n *= 800
± *s.d.*						
			
0.6	0.005	-0.002	0.002 ± 0.001	-0.002 ± 0.002	-0.002 ± 0.000	-0.002 ± 0.000
0.5	0.010	0.004	0.004 ± 0.001	0.004 ± 0.001	0.004 ± 0.000	0.004 ± 0.000
0.5	0.020	0.008	0.008 ± 0.002	0.008 ± 0.001	0.008 ± 0.001	0.008 ± 0.001
0.3	0.030	0.010	0.010 ± 0.003	0.011 ± 0.002	0.010 ± 0.002	0.010 ± 0.001
0.7	0.040	0.010	0.010 ± 0.004	0.011 ± 0.002	0.010 ± 0.002	0.010 ± 0.001
0.3	0.050	0.020	0.021 ± 0.005	0.021 ± 0.004	0.021 ± 0.003	0.021 ± 0.002
0.5	0.100	0.040	0.044 ± 0.008	0.045 ± 0.006	0.046 ± 0.004	0.045 ± 0.003
			
*LOD *± *s.d.*						
			
0.6	0.005	-0.002	1.852 ± 1.047	3.619 ± 1.467	6.913 ± 2.112	13.774 ± 2.927
0.5	0.010	0.004	1.949 ± 1.036	3.692 ± 1.454	7.397 ± 1.959	14.390 ± 2.809
0.5	0.020	0.008	2.010 ± 1.045	3.843 ± 1.483	7.657 ± 2.070	14.955 ± 2.832
0.3	0.030	0.010	1.545 ± 1.047	2.957 ± 1.395	5.596 ± 1.977	11.085 ± 2.717
0.7	0.040	0.010	1.115 ± 0.707	2.093 ± 1.043	3.958 ± 1.377	7.682 ± 2.063
0.3	0.050	0.020	2.325 ± 1.278	4.393 ± 1.690	8.648 ± 2.452	17.191 ± 3.332
0.5	0.100	0.040	2.493 ± 1.100	4.860 ± 1.549	9.560 ± 2.165	18.948 ± 2.994

Means and standard deviations of the estimates of the disequilibrium coefficients and the corresponding LOD score from using various sizes of case-control samples.

Table 6 lists the means and standard deviations of 1,000 repeated estimates of the disequilibrium coefficient and the corresponding LOD score values from the case-control samples of 200 individuals but with varying proportions of the cases and controls. Variation in the proportion of case individuals in the sample does not show observable influence on adequacy of estimates of the disequilibrium parameter but the LOD score values constantly increase with the increased proportion of controls.

**Table 6:** LD estimation from case and control samples with varying proportions

*p*	*q*	*D*	*c:c *= 3/4:1/4	*c:c*= 2/3:1/3	*c:c *= 1/3:2/3	*c:c *= 1/4:3/4
± *s.d.*						
			
0.6	0.005	-0.002	0.002 ± 0.000	-0.002 ± 0.000	-0.002 ± 0.000	-0.002 ± 0.000
0.5	0.010	0.004	0.004 ± 0.001	0.004 ± 0.001	0.004 ± 0.001	0.004 ± 0.001
0.5	0.020	0.008	0.008 ± 0.001	0.008 ± 0.001	0.008 ± 0.001	0.008 ± 0.002
0.3	0.030	0.010	0.010 ± 0.003	0.010 ± 0.003	0.010 ± 0.002	0.010 ± 0.003
0.7	0.040	0.010	0.010 ± 0.003	0.011 ± 0.002	0.010 ± 0.002	0.010 ± 0.003
0.3	0.050	0.020	0.021 ± 0.004	0.021 ± 0.004	0.021 ± 0.004	0.021 ± 0.004
0.5	0.100	0.040	0.043 ± 0.007	0.044 ± 0.006	0.045 ± 0.006	0.045 ± 0.007
			
*LOD *± *s.d.*						
			
0.6	0.005	-0.002	1.814 ± 0.855	2.420 ± 1.063	4.707 ± 2.172	5.362 ± 2.695
0.5	0.010	0.004	1.906 ± 0.832	2.493 ± 0.997	4.915 ± 1.994	5.715 ± 2.502
0.5	0.020	0.008	1.969 ± 0.812	2.579 ± 1.029	5.112 ± 2.160	5.916 ± 2.653
0.3	0.030	0.010	1.481 ± 0.812	1.950 ± 1.025	3.888 ± 2.011	4.463 ± 2.519
0.7	0.040	0.010	1.063 ± 0.580	1.453 ± 0.724	2.714 ± 1.368	3.130 ± 1.783
0.3	0.050	0.020	2.162 ± 0.966	2.982 ± 1.161	5.876 ± 2.446	6.695 ± 3.029
0.5	0.100	0.040	2.275 ± 0.816	3.152 ± 1.071	6.657 ± 2.241	7.616 ± 2.772

Means and standard deviations of the estimates of the disequilibrium coefficients and the corresponding LOD score from use of case-control samples of a constant size of 200 individuals but with varying proportions between the 'case and control' individuals, *c:c*.

### Analysis of β-thalassemia dataset

*β*-thalassemia is an autosomal recessive hemoglobinopathy caused by mutations in the *β*-globin (HBB) gene (OMIM 141900). The disorder is one of the most common inherited hemoglobinopathies in the world, with estimates of carrier frequencies ranging from 3 to 10% in some areas of the tropics and subtropics including southern China [19,21,22]. A frame shift mutation in codons 41 and 42, a 4-bp deletion (-CTTT), of the human *β*-globin gene represents the most common *β*-thalassemia mutations in East and Southeast Asia. The population frequency of the deletion is as high as 3% in South China [19]. To survey the distribution of linkage disequilibrium among the polymorphic sites surrounding the *β*-globin gene, Zhang et al collected a sample of 40 Chinese individuals, including 16 *β*^CD41/42 ^thalassemia heterozygotes and 24 normal individuals [19]. They directly sequenced a 15.933-kb DNA region spanning 20.693 kb of the *β*-globin cluster surrounding the deletion and detected 50 bi-allelic sites in the sequenced region. All individuals in the sample were genotyped at the polymorphic markers. This dataset represents a typical example of the selected sample in which disease carriers are deliberately enriched and no homozygote of the disease allele (i.e. at the deletion locus) is present in the sample.

**Figure 1:**
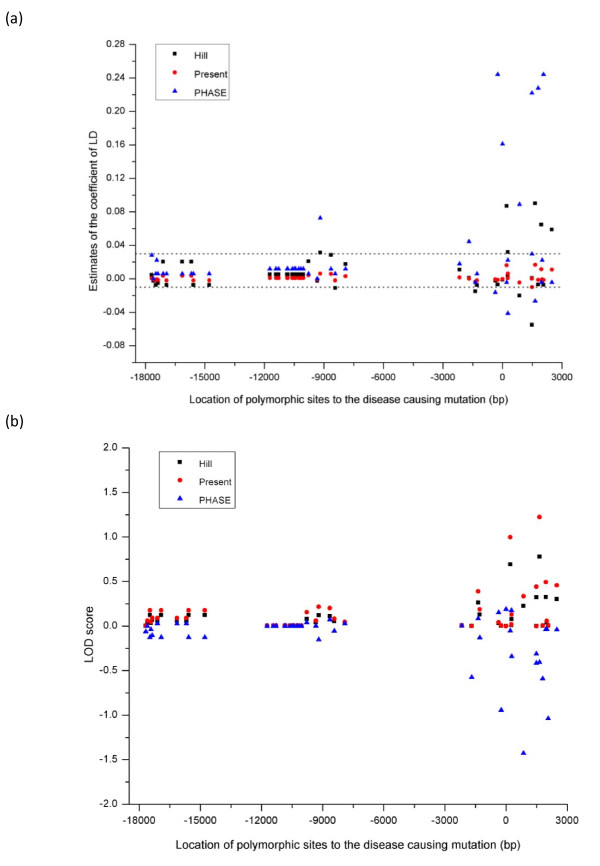
**Linkage disequilibrium analysis of the *β-thalassemia *sample**. Distribution of linkage disequilibrium between each of polymorphic sites and the *β-thalassemia *causing mutation in a 20.693 kb region surrounding the human *β *-globin gene. (a) Estimates of the coefficients of LD from three different methods. The dot lines represent the lowest and highest theoretical bounds of the disequilibrium parameter, which are defined by allele frequencies at the polymorphic marker and disease causing sites. (b) The LOD score values calculated for the LD estimates from the three methods.

To evaluate linkage disequilibria across the sequenced region, the haplotypes in the sample were first predicted by use of the computer software PHASE2.1.1 developed by Stephens et al [10], and the predicted haplotypes were then used to calculate the coefficients of linkage disequilibrium. We implemented the haplotype-based method and the two methods (*H *and *L*) to analyze the sequence data and calculated the coefficient of linkage disequilibrium between the disease mutation, which was set as position 0, and each of the other polymorphic sites. We used the population frequency of 3% for the disease causing allele in the analysis and illustrate the distribution of estimates of the disequilibrium between each of the polymorphic sites and the disease causing mutation in Figure 1(a) together with the lowest and highest bounds for the disequilibrium parameter which were inferred from the maker allele frequency estimates and the population frequency of the disease causing allele. It can be seen that the LD estimates from the present method could differ substantially from the other two methods. Importantly, the LD estimates by the former lie properly between the corresponding theoretical bounds whilst the estimates by the latter two may be severely out of the bounds, suggesting serious bias in the estimates to the true parameters. Figure 1(b) shows the LOD score values for the LD estimates from the three methods and demonstrates that the present method provides much more likely estimates of linkage disequilibria in the vicinity of the disease causing site than the other methods under comparison. The data analysis clearly indicates that the method developed here is robust to non-randomness of the samples by which linkage disequilibrium is statistically inferred.

## Discussion

The past decade has witnessed great progress in high-throughput detection and genotyping of single nucleotide polymorphisms (SNPs) in the genomes of plants, animals and humans. This has stimulated tremendous interest in mapping and identifying subtle genetic variants contributing to phenotypic variation of complex traits in natural populations through detecting linkage disequilibrium maintained in the populations by close linkage between alleles at genetic polymorphic sites and at trait loci. However, a common concern of association studies is the high proportion of false positive or false negative tests of association of trait phenotype with causal genetic polymorphisms as well as the limited statistical power in detecting genuine associations [23,24]. A rich pool of literature has been focused on exploring factors that cause these problems and seeking for solutions to them. The most prominent among these is population stratification that could cause both false positive and false negative inferences of association [25].

Although it has been well established that skewed sampling from a population with a linkage equilibrium distribution of multi-locus genotypes may result in spurious linkage disequilibrium [26], there has not been a comprehensive investigation of the consequences of non-random samples on statistical inference of LD from populations under disequilibrium. We demonstrate in the present study that the use of non-random samples could result in severely biased estimates of LD from the method proposed for random samples. The simulation study showed that the estimates could be so biased as to be outside the theoretical limits of the corresponding simulated values and the biases can be either up- or downwards. These results indicate that the non-randomness of the sample may result in considerable false positive or false negative inference of the disequilibrium parameter and, in turn, false positives or false negatives in association analyses in which significant degree of marker-disease association implies significant linkage disequilibrium. Instead of considering the joint probability distribution of genotypes at the marker and disease loci in estimating LD from random samples [6,13], we propose the use of the conditional probability distribution of disease genotypes given any marker genotype in developing a new method to estimate the parameter. The method avoids or effectively alleviates the influence of non-random presentation of any marker-disease genotype in the samples on the parameter estimation. On the basis of simulation studies, we show that the method yields equally adequate estimates of LD to the method previously proposed [6] and currently widely cited in the literature when individual genotypes are randomly sampled from the populations. However, the method confers significant improvement over the current method when the parameter estimation is made from using artificially selected samples. Methodologically, the improvement in the parameter estimation of the method developed in the present study over the current methods [6,13] can be explained by their difference in extracting information of linkage disequilibrium between the two loci of interest from the samples under study. The present method uses the conditional probability distribution of genotypes at one locus on any given genotype at the other locus as illustrated in Table 1. When the sample of study is collected in such a way that genotype(s) at one of the two loci undergo selection, for example, the case-control samples where genotypes at the disease locus are strongly selected, the joint distribution of genotypes at the two loci in the sample deviates greatly from the joint genotypic distribution in the population from which the sample is collected. However, the conditional distribution of genotypes at one locus given any genotype at the other selected or unselected locus in the sample remains approximately the same as that in the population. Hence the analysis based on the conditional genotypic distribution is more robust to non-randomness of the samples to infer linkage disequilibrium in populations of interest than that based on the joint genotypic distribution.

The 'case and control' design used in many association studies to screen for genetic polymorphisms in significant association with phenotypic variation probably illustrates the most popular example of LD analysis with non-random samples [11,12,14-18]. Although the base populations under investigation may be randomly mating with respect to genotypes at marker-disease loci, the samples used in the studies are collected so that case individuals are well represented and thus create severely non-random presentation of the populations. Given that the analysis of the samples is to infer the situation in the base populations, accurate inference of LD from the samples is obviously crucial for reliability of these analyses. The *β-thalassemia *data analysis in the present study represents a typical example of such studies. The analysis with the dataset, although very limited in sample size, highlights the importance of adequately tackling the non-randomness in the 'case and control' studies in order to achieve reliable assessment of LD. It clearly demonstrates the robustness of the method developed in the present study to the non-randomness. Previous methods, which are widely implemented in the current literature of 'case and control' studies are only suitable for analysis with random samples and so can result in seriously biased inference of the disequilibrium parameter.

**Figure 2:**
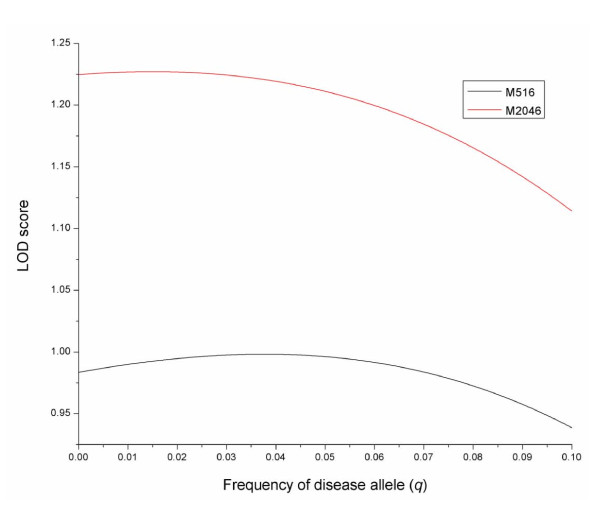
**LOD score profile**. LOD score profiles for the MLE of linkage disequilibrium between the disaese causing mutant and each of two linked polymorphic markers M516 and M2046 over different values of disease allele frequency. The two markers are of 516 and 2046 bp to the disease causing mutant site.

A practical problem that is raised to accurately infer LD from 'case and control' samples is the need for accurate estimates of allelic frequencies at both marker and putative disease loci. We propose the use of control samples to calculate marker allelic frequency because there is usually not any prior selective criterion imposed on the marker genotypes of those individuals to be included in the control samples. It is shown that accurate estimates of the allele frequency are the basis for accurate estimation of the disequilibrium parameter (Table 4), but neither the 'cases' or 'controls' separately or together are likely appropriate to estimate the allele frequency at the putative disease locus. In the scenario where epidemiological information is available for disease candidates such as the *β-thalassemia *data considered in the present study, the allele frequency estimate from a prior population survey may be used to meet this need. Alternatively, one can search the LOD score profile (equation 1) for the MLE of the disequilibrium parameter over various *q *values and choose the MLE of *D *calculated from the *q *value at which the likelihood profile reaches the maximum. Figure 2 illustrates such an example with the *β-thalassemia *data analysis and shows that the MLE of *D *was obtained when *q *took a value of ~3%, which is near to the estimate from the previous population survey [19].

Methodologically, the present study has been focused on the most prominent linkage disequilibrium measure, *D *as defined above. There are several other measures, such as *D' *or *r*^2 ^etc, frequently used in the literature of genetic association study or population genetic analysis. These latter measures are either a scaled or standardized form of the basic disequilibrium parameter *D*. Bias in estimate of *D *will be inherent to that of transformed versions of the parameter. For example, we re-analyzed the *β-thalassemia *data by using *r*^2^, which is defined as *D*^2^/*p*(1-*p*)*q*(1-*q*) in the present notation, as the disequilibrium measure and showed the analysis in Additional file 3. The disequilibrium distribution shows an almost identical pattern to that demonstrated in Figure 1, in which *D *was the disequilibrium measure.

One of the key properties of 'case and control' designs is its statistical power in detecting association between a genetic polymorphic marker to the phenotype of a disease trait. It has been widely accepted that level of LD is a critical factor in determining the power of 'case and control' designs to detect significance of genetic association [27,28]. Several studies have focused on investigating factors affecting statistical power of the 'case and control' design, including marker allele frequency and errors in marker genotype and trait phenotype [29-31]. An equal proportion of cases and controls were proposed in these studies and implemented in many real 'case and control' experimental analyses [11,31]. The present study investigated the impact of using varying proportions of the cases and controls on the power of the 'case and control' design in detecting linkage disequilibrium and shows that, for a given size of the 'case and control' sample, increasing the proportion of the 'cases' decreases rather than increases the statistical power (Table 6). In fact, increasing the proportion of control individuals in the 'case and control' samples alleviates the effects of non-randomness of the samples. This result indicates a need of reconsideration of the commonly accepted sampling strategy for 'case and control' study designs. We re-examined the question by implementing the chi-square based test proposed to test for significance of difference in marker allele frequency between cases and controls. The analysis was summarized as an Additional file 4 and shows that use of an equal proportion of cases and controls in the case-control design is favoured for a higher statistical power to detect the genetic associations. However, it has been well established that the chi-square based association test is highly vulnerable to deviation of genotypic distribution from the Hardy-Weinberg equilibrium [32]. Any violation to the equilibrium may result in severe type I error. Moreover, it is clear from Additional file 2 that the equilibrium does not usually hold in these samples.

It is well established that LD based association analysis is effective only for the genes underlying Mendelian traits or the genes with major genetic effects on polygenic traits [23,33]. In the present study we assumed that genotypes at the putative disease locus are observable as are those at the marker locus. The assumption holds for Mendelian traits but may be questionable for quantitative traits. In fact, the theoretical model and analysis developed in the present study can be incorporated into the statistical framework we previously developed for detecting and estimating linkage disequilibrium between a genetic marker and a locus affecting a quantitative trait showing continuous or dichotomous phenotypic variation [20,34,35]. The quantitative genetic model allows allelic frequencies at the marker and trait loci, the coefficient of linkage disequilibrium between the two loci, genetic effects at the trait locus and the residual variance component to be modelled and inferred. Integration of the two models enables the non-randomness of samples to be properly accounted for in the statistical inference on the genetic parameters.

## Conclusions

We demonstrated that the non-randomness may cause seriously biased assessment of LD when using the current methods originally developed for random samples. We have developed a new approach for inferring LD from samples with various degrees of non-randomness, and showed the significantly improved robustness of the present approach over the current methods when non-random samples were to evaluate LD through intensive simulation studies and analysis of a case and control sample of *β*-thalasemia. As accurate estimation of the disequilibrium parameter is crucial for any association study, in which the case/control design represents a typical example of non-random samples, the present paper highlights the importance of tackling the problem of using non-random samples to the community of LD analysis, and in addition, provides a route to improve statistical reliability in association studies.

## Methods

**Inferring LD from Using Nonrandom Samples**: we consider two biallelic loci M (marker locus) and A (disease locus). There are two alleles at the marker locus, *M *and *m*, with population frequencies *p *and 1-*p *respectively and two alleles at the disease locus, *A *and *a*, with population frequencies *q *and 1-*q*. For simplicity, but without loss of generality, we denote by *D*, the coefficient of linkage disequilibrium between genes at the two loci. The conditional probability (*f*_*ij*_) of genotypes at one of two loci given a genotype at the other locus can be expressed in term of these genetic parameters when assuming random mating in the population. For example, the disease genotype distribution given any marker genotype is presented in Table 1.

Among a random sample collected from such a random mating population, let *n*_*ij *_be the number of individuals with the *j*th (*j *= 1, 2, 3) genotype at the disease locus and the *i*th (*i *= 1, 2, 3) genotype at the marker locus. The logarithm of the likelihood function of the genetic parameters, *p*, *q *and *D*, given the observations *n*_*ij *_has the form:(1)

Although there are three parameters in the log-likelihood function, our focus is on *D*, the coefficient of linkage disequilibrium. We will investigate and discuss impacts of the other two parameters, *p *and *q*, on statistical inference on *D*. The maximum likelihood estimation (MLE) of *D *can be calculated from the equation

which can be solved explicitly by using *Mathematica *[36] and from a polynomial equation with power of 5(2)

Mathematical forms for *a*_*i *_(*i *= 0, 1, 2, ..., 5) are given in Additional file 5 (on the website of *BMC Genomics*). Although an analytical solution does not exist, equation (2) can be solved numerically [37]. The equation has at least one real root because *a*_5 _= 4*n *> 0 where *n *is the sample size. If multiple real roots were found, we determined , the maximum likelihood estimate of *D*, by comparing their corresponding likelihood values. It should be noticed that a meaningful estimate of *D *must not be beyond its theoretical bounds {*max*(-*pq*,-(1 - *p*)(1 - *q*),*min*(*p*(1 - *q*),(1 - *p*)*q*)}. Thus, the MLEs of the parameter were chosen from possible roots of the equation (2) that lies in the theoretical bounds and corresponds to the highest likelihood. With the MLE and the likelihood function, one can test for significance of the disequilibrium parameter using the likelihood ratio test statistic(3)

which asymptotically follows a chi-square distribution with 1 degree of freedom. It should be noticed that the above analysis is based on the conditional probability distribution of genotypes at one locus given any genotype at the other of the two loci.

**Inferring LD from Using Random Samples**: By assuming use of samples randomly collected from a randomly mating population, Hill (1974) proposed estimating the MLE of frequency of haplotype *MA*, say *g*_11_, through an iterative procedure, which is well known as the "chromosome counting" algorithm in the literature [6]. Mano et al highlighted that the iterative algorithm may lead to local maxima and proposed conditions for their existence [38]. Weir and Cockerham [13] showed that a more robust approach to calculate the MLE of *g*_11 _is to solve it from the cubic equation given below(4)

where *b*_0 _= -(2*n*_11 _+ *n*_12 _+ *n*_21_)*pq*, *b*_1 _= 2*npq *- (2*n*_11 _+ *n*_12 _+ *n*_21_)(1 - 2*p *- 2*q*) - *n*_22_(1 - *p *- *q*), *b*_2 _= 2*n*(1 - 2*p *- 2*q*) - 2(2*n*_11 _+ *n*_12 _+ *n*_21_) - *n*_22 _and *b*_3 _= 2*n*. With ,  and  the MLE of *g*_11_, *p *and *q *respectively, the MLE of the coefficient of disequilibrium coefficient, *D*, can be calculated as . Weir and Cockerham stressed that calculation of  by solving the cubic equation may avoid the scenario of the iterative procedure may be diverging or converging but to the wrong roots [13]. Because *b*_3 _= 2*n *< 0, the cubic must have at least one real root. The theoretical bounds for *g*_11 _are given by {*max*(0, *p *+ *q *- 1), *min*(*p*,*q*)} [13]. Significance of the MLE of *D *can also be tested through a likelihood ratio test statistic with a form similar to equation (3). However, to evaluate the likelihood function, one needs to use the joint genotype probability distribution given by Hill [6]. It should be noticed that the above analysis is based on the joint probability distribution of genotypes at the two loci of interest.

The above analyses show that the disequilibrium parameter can be estimated and detected from the two methods. In the **Results **section, we explored the performance of these methods at inferring the linkage disequilibrium using a simulation study and by analysis of real data.

## Authors' contributions

ZL conceived of the study and developed the theoretical analyses. MW conducted the simulation study and analyzed the *β*-thalassemia dataset, with assistance from TJ, NJ and LW. MW and ZL wrote the paper. All authors read and approved the final manuscript.

## Supplementary Material

Additional file 1 Table S1 Simulation of Sampling Scheme II using larger sample sizes. Click here for file

Additional file 2 Table S2 Hardy-Weinberg equilibrium test. Click here for file

Additional file 3 Figure S1 Linkage disequilibrium measured in *r*^2^. Click here for file

Additional file 4 Table S3 Chi-square test of case and control samples with varying proportions. Click here for file

Additional file 5 Mathematical forms for the coefficients of the polynomial equation (2). Click here for file
