# Supplementary material for: Inferring linkage disequilibrium from non-random samples†
Source: BMC Genomics. 2010 May 26;11:328. doi: 10.1186/1471-2164-11-328 (PMC2890561; doi:10.1186/1471-2164-11-328)
Supplement: Additional file 1 — Table S1 Simulation of Sampling Scheme II using larger sample sizes. [file 1471-2164-11-328-S1.DOC]

**Table S1** **Simulation of Sampling Scheme II using larger sample sizes.** Means and standard deviations of 1,000 estimated coefficients of linkage disequilibrium from Methods H and L when *n* individuals were generated from the Sampling Scheme II in which either a marker genotype ( = 0, *i* = 1,2,3) or a marker-disease genotype ($n_{ii}$= 0, *i* = 1,2,3) was missing. The population numbers are the same as those in Table 2 and *D* is the true value of the disequilibrium coefficient. (a) *n*=400, (b) *n*=800.

(a)

| Pop. | *D* | *.* = 0 | | *.* = 0 | | = 0 | | *n_11_* = 0 | | *n_22_* = 0 | | *n_33_* = 0 | |
| --- | --- | --- | --- | --- | --- | --- | --- | --- | --- | --- | --- | --- | --- |
|  |  | $\hat{D}_{H}\pm s.d.$ | $\hat{D}_{L}\pm s.d.$ | $\hat{D}_{H}\pm s.d.$ | $\hat{D}_{L}\pm s.d.$ | $\hat{D}_{H}\pm s.d.$ | $\hat{D}_{L}\pm s.d.$ | $\hat{D}_{H}\pm s.d.$ | $\hat{D}_{L}\pm s.d.$ | $\hat{D}_{H}\pm s.d.$ | $\hat{D}_{L}\pm s.d.$ | $\hat{D}_{H}\pm s.d.$ | $\hat{D}_{L}\pm s.d.$ |
| 1 | 0.20 | 0.18±0.01 | 0.20±0.01 | 0.20±0.01 | 0.20±0.01 | 0.18±0.01 | 0.20±0.01 | 0.17±0.01 | 0.18±0.01 | 0.17±0.01 | 0.17±0.01 | 0.17±0.01 | 0.18±0.01 |
| 2 | 0.10 | 0.09±0.01 | 0.10±0.01 | 0.10±0.01 | 0.10±0.01 | 0.09±0.01 | 0.10±0.01 | 0.05±0.01 | 0.06±0.01 | 0.07±0.01 | 0.07±0.01 | 0.05±0.01 | 0.06±0.01 |
| 3 | 0.09 | 0.08±0.01 | 0.09±0.01 | 0.06±0.01 | 0.09±0.01 | 0.10±0.01 | 0.09±0.01 | 0.07±0.01 | 0.08±0.01 | 0.04±0.01 | 0.06±0.01 | 0.00±0.02 | 0.04±0.01 |
| 4 | 0.09 | 0.10±0.01 | 0.09±0.01 | 0.06±0.01 | 0.09±0.01 | 0.08±0.01 | 0.09±0.01 | 0.00±0.02 | 0.04±0.01 | 0.04±0.01 | 0.06±0.01 | 0.07±0.01 | 0.08±0.01 |
| 5 | 0.10 | 0.08±0.01 | 0.10±0.01 | 0.06±0.01 | 0.10±0.01 | 0.11±0.01 | 0.10±0.01 | 0.07±0.01 | 0.08±0.01 | 0.07±0.01 | 0.08±0.01 | 0.05±0.01 | 0.06±0.01 |
| 6 | 0.10 | 0.09±0.01 | 0.10±0.01 | 0.10±0.01 | 0.10±0.01 | 0.09±0.01 | 0.10±0.01 | 0.07±0.01 | 0.08±0.01 | 0.07±0.01 | 0.08±0.01 | 0.05±0.01 | 0.06±0.01 |

(b)

| Pop. | *D* | *.* = 0 | | *.* = 0 | | = 0 | | *n_11_* = 0 | | *n_22_* = 0 | | *n_33_* = 0 | |
| --- | --- | --- | --- | --- | --- | --- | --- | --- | --- | --- | --- | --- | --- |
|  |  | $\hat{D}_{H}\pm s.d.$ | $\hat{D}_{L}\pm s.d.$ | $\hat{D}_{H}\pm s.d.$ | $\hat{D}_{L}\pm s.d.$ | $\hat{D}_{H}\pm s.d.$ | $\hat{D}_{L}\pm s.d.$ | $\hat{D}_{H}\pm s.d.$ | $\hat{D}_{L}\pm s.d.$ | $\hat{D}_{H}\pm s.d.$ | $\hat{D}_{L}\pm s.d.$ | $\hat{D}_{H}\pm s.d.$ | $\hat{D}_{L}\pm s.d.$ |
| 1 | 0.20 | 0.18±0.00 | 0.20±0.00 | 0.20±0.00 | 0.20±0.00 | 0.18±0.00 | 0.20±0.00 | 0.17±0.00 | 0.18±0.00 | 0.17±0.00 | 0.17±0.00 | 0.17±0.00 | 0.18±0.00 |
| 2 | 0.10 | 0.09±0.01 | 0.10±0.01 | 0.10±0.01 | 0.10±0.01 | 0.09±0.01 | 0.10±0.01 | 0.05±0.01 | 0.06±0.01 | 0.07±0.01 | 0.07±0.01 | 0.05±0.01 | 0.06±0.01 |
| 3 | 0.09 | 0.08±0.01 | 0.09±0.01 | 0.06±0.01 | 0.09±0.01 | 0.10±0.01 | 0.09±0.01 | 0.07±0.01 | 0.08±0.01 | 0.04±0.01 | 0.06±0.01 | 0.00±0.01 | 0.04±0.01 |
| 4 | 0.09 | 0.10±0.01 | 0.09±0.01 | 0.06±0.01 | 0.09±0.01 | 0.08±0.01 | 0.09±0.01 | 0.00±0.01 | 0.04±0.01 | 0.04±0.01 | 0.06±0.01 | 0.07±0.01 | 0.08±0.01 |
| 5 | 0.10 | 0.08±0.01 | 0.10±0.01 | 0.06±0.01 | 0.10±0.01 | 0.12±0.01 | 0.10±0.00 | 0.07±0.01 | 0.08±0.01 | 0.07±0.00 | 0.08±0.00 | 0.05±0.01 | 0.06±0.01 |
| 6 | 0.10 | 0.09±0.01 | 0.10±0.01 | 0.10±0.01 | 0.10±0.00 | 0.09±0.01 | 0.10±0.01 | 0.07±0.01 | 0.08±0.01 | 0.07±0.00 | 0.08±0.01 | 0.05±0.01 | 0.06±0.01 |
