# Supplementary material for: Inferring linkage disequilibrium from non-random samples†
Source: BMC Genomics. 2010 May 26;11:328. doi: 10.1186/1471-2164-11-328 (PMC2890561; doi:10.1186/1471-2164-11-328)
Supplement: Additional file 2 — Table S2 Hardy-Weinberg equilibrium test. [file 1471-2164-11-328-S2.DOC]

**Table S2 Hardy-Weinberg equilibrium test.** Levels of probability of the test for significance of Hardy-Weinberg equilibrium at marker (M) and disease (A) loci. The population numbers are the same as those in Table 3.

| Pop. | *.* = 0 | | *.* = 0 | | = 0 | | *n_11_* = 0 | | *n_22_* = 0 | | *n_33_* = 0 | |
| --- | --- | --- | --- | --- | --- | --- | --- | --- | --- | --- | --- | --- |
|  | M | A | M | A | M | A | M | A | M | A | M | A |
| 1 | 0.00E+00 | 0.00E+00 | 0.00E+00 | 0.00E+00 | 0.00E+00 | 0.00E+00 | 0.00E+00 | 0.00E+00 | 0.00E+00 | 0.00E+00 | 0.00E+00 | 0.00E+00 |
| 2 | 0.00E+00 | 1.03E-05 | 0.00E+00 | 1.01E-03 | 0.00E+00 | 4.00E-05 | 9.80E-08 | 3.78E-08 | 0.00E+00 | 0.00E+00 | 2.11E-07 | 3.15E-08 |
| 3 | 0.00E+00 | 1.86E-02 | 0.00E+00 | 1.90E-06 | 0.00E+00 | 0.00E+00 | 1.25E-02 | 1.30E-02 | 0.00E+00 | 0.00E+00 | 0.00E+00 | 0.00E+00 |
| 4 | 0.00E+00 | 0.00E+00 | 0.00E+00 | 1.34E-06 | 0.00E+00 | 2.12E-02 | 0.00E+00 | 0.00E+00 | 0.00E+00 | 0.00E+00 | 1.50E-02 | 1.22E-02 |
| 5 | 0.00E+00 | 2.17E-02 | 0.00E+00 | 1.49E-06 | 0.00E+00 | 0.00E+00 | 2.51E-07 | 9.96E-03 | 0.00E+00 | 0.00E+00 | 3.82E-07 | 0.00E+00 |
| 6 | 0.00E+00 | 1.06E-06 | 0.00E+00 | 1.37E-04 | 0.00E+00 | 3.49E-06 | 1.14E-02 | 7.22E-07 | 0.00E+00 | 0.00E+00 | 0.00E+00 | 6.92E-08 |
