# Supplementary material for: Inferring linkage disequilibrium from non-random samples†
Source: BMC Genomics. 2010 May 26;11:328. doi: 10.1186/1471-2164-11-328 (PMC2890561; doi:10.1186/1471-2164-11-328)
Supplement: Additional file 3 — Figure S1 Linkage disequilibrium measured in r2. [file 1471-2164-11-328-S3.DOC]

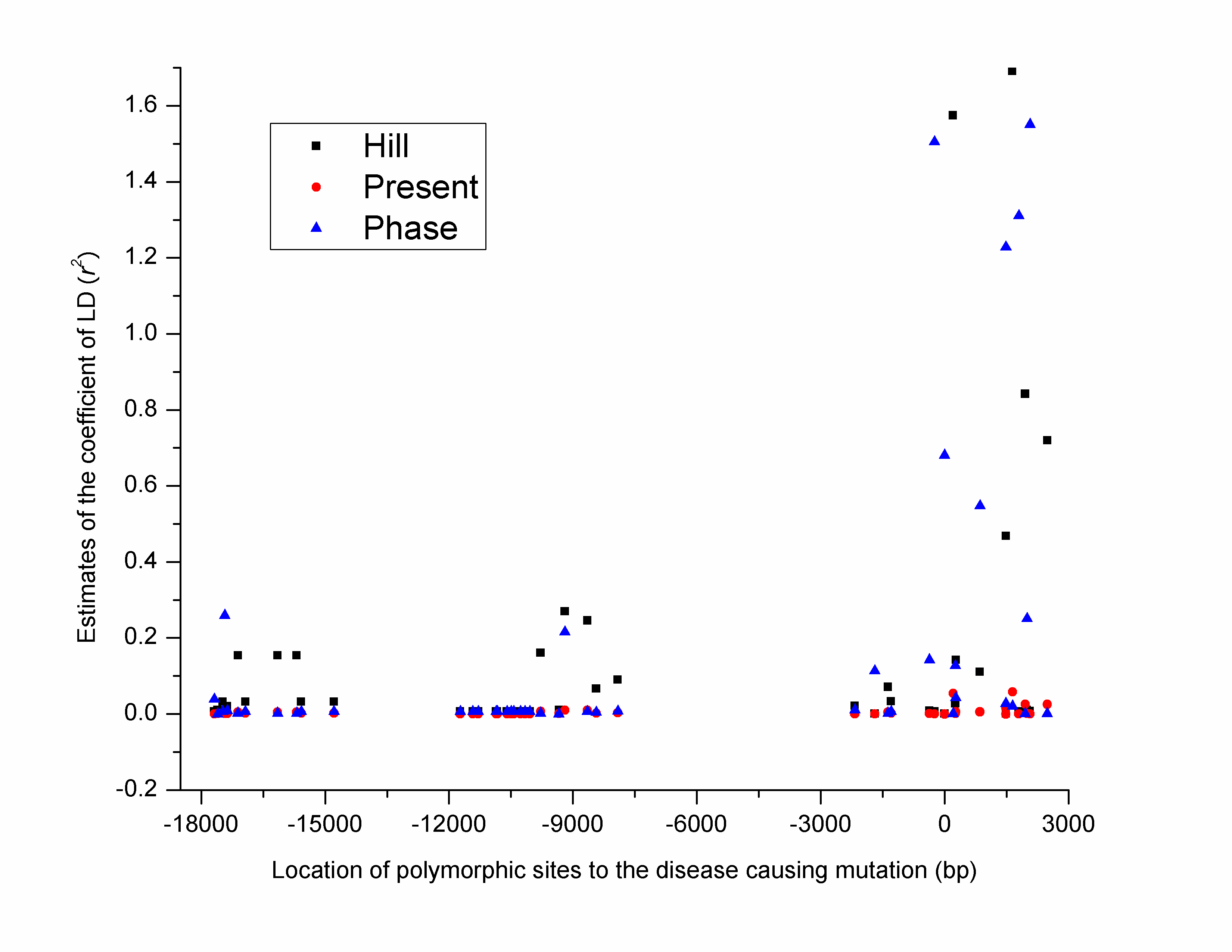


**Figure S1 Linkage disequilibrium measured in term of *r^2^*.** Distribution of linkage disequilibrium measured by *r^2^* between each of polymorphic sites and the *β-thalassemia* causing mutation in a 20.693 kb region surrounding the human *β* -globin gene.
