# Supplementary material for: Inferring linkage disequilibrium from non-random samples†
Source: BMC Genomics. 2010 May 26;11:328. doi: 10.1186/1471-2164-11-328 (PMC2890561; doi:10.1186/1471-2164-11-328)
Supplement: Additional file 4 — Table S3 Chi-square test of case and control samples with varying proportions. [file 1471-2164-11-328-S4.DOC]

**Table S3 Chi-square test of case and control samples with varying proportion.** Means and standard deviations of the Pearson’s chi-square test statistics ($\chi_{[1]}^{2}$) and the corresponding *P* values for the means of $\chi_{[1]}^{2}$ from case-control samples of a constant size of 200 individuals but with varying proportions between the ‘case and control’ individuals, *c:c*.

| *p* | *q* | *D* | *c:c* = 3/4:1/4 | | *c:c* = 2/3:1/3 | | *c:c* = 1/2:1/2 | | *c:c* = 1/3:2/3 | | *c:c* = 1/4:3/4 | |
| --- | --- | --- | --- | --- | --- | --- | --- | --- | --- | --- | --- | --- |
|  |  |  | $\chi_{[1]}^{2}\pm s.d.$ | *P* value | $\chi_{[1]}^{2}\pm s.d.$ | *P* value | $\chi_{[1]}^{2}\pm s.d.$ | *P* value | $\chi_{[1]}^{2}\pm s.d.$ | *P* value | $\chi_{[1]}^{2}\pm s.d.$ | *P* value |
| 0.6 | 0.005 | -0.002 | 13.3±6.9 | 2.70×10^-4^ | 15.7±7.9 | 7.50×10^-5^ | 16.8±7.4 | 4.20×10^-5^ | 15.3±7.0 | 9.00×10^-5^ | 13.5±6.6 | 2.40×10^-4^ |
| 0.5 | 0.01 | 0.004 | 14.9±7.6 | 1.10×10^-4^ | 17.0±7.9 | 3.70×10^-5^ | 17.8±7.8 | 2.40×10^-5^ | 15.6±7.0 | 7.70×10^-5^ | 13.3±6.0 | 2.60×10^-4^ |
| 0.5 | 0.02 | 0.008 | 15.1±7.6 | 1.00×10^-4^ | 17.3±7.5 | 3.30×10^-5^ | 18.6±7.9 | 1.60×10^-5^ | 16.1±7.0 | 6.10×10^-5^ | 13.4±6.3 | 2.60×10^-4^ |
| 0.3 | 0.03 | 0.01 | 10.3±5.8 | 1.30×10^-3^ | 12.0±6.4 | 5.20×10^-4^ | 13.9±6.9 | 2.00×10^-4^ | 13.0±6.8 | 3.10×10^-4^ | 11.2±6.4 | 8.10×10^-4^ |
| 0.7 | 0.04 | 0.01 | 9.2±6.1 | 2.40×10^-3^ | 10.5±6.4 | 1.20×10^-3^ | 10.6±6.1 | 1.10×10^-3^ | 8.5±4.9 | 3.50×10^-3^ | 7.4±4.5 | 6.70×10^-3^ |
| 0.3 | 0.05 | 0.02 | 15.2±7.2 | 9.90×10^-5^ | 17.8±7.4 | 2.50×10^-5^ | 20.5±8.5 | 6.10×10^-6^ | 19.0±8.2 | 1.30×10^-5^ | 16.6±7.7 | 4.70×10^-5^ |
| 0.5 | 0.1 | 0.04 | 18.5±8.6 | 1.70×10^-5^ | 21.6±8.8 | 3.30×10^-6^ | 23.5±8.4 | 1.20×10^-6^ | 20.4±7.9 | 6.20×10^-6^ | 16.9±7.2 | 3.90×10^-5^ |

Case-control sample of size 200 individuals with varying proportions of cases is generated and summarized according to marker genotype and case/control status as

|  | *Mm* | *Mm* | *mm* | Sum |
| --- | --- | --- | --- | --- |
| Case | *n_11_* | *n_12_* | *n_13_* | *n_1_* |
| Control | *n_21_* | *n_22_* | *n_23_* | *n_2_* |

Given the above contingency table, Pearson’s chi-square test statistic is formulated by

$$\chi_{[1]}^{2}=\frac{(\hat{p}_{D}-\hat{p}_{C})^{2}}{\hat{p}(1-\hat{p})\left(\frac{1}{2n_{1}}+\frac{1}{2n_{2}}\right)}$$

where $\hat{p}_{D}=(2n_{11}+n_{12})/(2n_{1})$,$\hat{p}_{C}=(2n_{21}+n_{22})/(2n_{2})$and$\hat{p}=(2n_{11}+n_{12}+2n_{21}+n_{22})/(2n)$.
