# Supplementary material for: Inferring linkage disequilibrium from non-random samples†
Source: BMC Genomics. 2010 May 26;11:328. doi: 10.1186/1471-2164-11-328 (PMC2890561; doi:10.1186/1471-2164-11-328)
Supplement: Additional file 5 — Mathematical forms for the coefficients of the polynomial equation (2). [file 1471-2164-11-328-S5.DOC]

**Additional file 5** Mathematical forms for the coefficients of the polynomial equation (2)

$$a_{5}=4n$$

$$\begin{matrix} a_{4}=6n_{11}+6n_{12}+6n_{13}+6n_{21}+5n_{22}+4n_{23}+6n_{31}+4n_{32}+2n_{33}+(-12n_{11}-12n_{12}- \\ 12n_{13}-10n_{21}-10n_{22}-10n_{23}-8n_{31}-8n_{32}-8n_{33})q-p\left[(12n_{11}+10n_{12}+8n_{13}+\right. \\ 12n_{21}+10n_{22}+8n_{23}+12n_{31}+10n_{32}+8n_{33}-(20n_{11}+20n_{12}+20n_{13}+20n_{21}+ \\ \left.20n_{22}+20n_{23}+20n_{31}+20n_{32}+20n_{33})q\right] \end{matrix}$$

$$\begin{matrix} a_{3}=2n_{11}+2n_{12}+2n_{13}+2n_{21}+n_{22}+n_{23}+2n_{31}+n_{32}- \\ (12n_{11}+12n_{12}+12n_{13}+9n_{21}+8n_{22}+7n_{23}+6n_{31}+4n_{32}+2n_{33})q+ \\ (12n_{11}+12n_{12}+12n_{13}+8n_{21}+8n_{22}+8n_{23}+4n_{31}+4n_{32}+4n_{33})q^{2}- \\ p\left[12n_{11}+9n_{12}+6n_{13}+12n_{21}+8n_{22}+4n_{23}+12n_{31}+7n_{32}+2n_{33}-\right. \\ (54n_{11}+48n_{12}+42n_{13}+48n_{21}+40n_{22}+32n_{23}+42n_{31}+32n_{32}+22n_{33})q- \\ \left.(48n_{11}+48n_{12}+48n_{13}+40n_{21}+40n_{22}+40n_{23}+32n_{31}+32n_{32}+32n_{33})q^{2}\right] \\ +p^{2}\left[12n_{11}+8n_{12}+4n_{13}+12n_{21}+8n_{22}+4n_{23}+12n_{31}+8n_{32}+4n_{33}-\right. \\ (48n_{11}+40n_{12}+32n_{13}+48n_{21}+40n_{22}+32n_{23}+48n_{31}+40n_{32}+32n_{33})q+ \\ \left.(40n_{11}+40n_{12}+40n_{13}+40n_{21}+40n_{22}+40n_{23}+40n_{31}+40n_{32}+40n_{33})q^{2}\right] \end{matrix}$$

$$\begin{matrix} a_{2}=-(2n_{11}+2n_{12}+2n_{13}+n_{21}+n_{22}+n_{23})q+ \\ 3(2n_{11}+2n_{12}+2n_{13}+n_{21}+n_{22}+n_{23})q^{2}-2(2n_{11}+2n_{12}+2n_{13}+n_{21}+n_{22}+n_{23})q^{3}- \\ p\left[2n_{11}+n_{12}+2n_{21}+n_{22}+2n_{31}+n_{32}+\right. \\ (26n_{11}+20n_{12}+14n_{13}+20n_{21}+14n_{22}+8n_{23}+14n_{31}+8n_{32}+2n_{33})q- \\ (60n_{11}+54n_{12}+48n_{13}+42n_{21}+36n_{22}+30n_{23}+24n_{31}+18n_{32}+12n_{33})q^{2}+ \\ \left.(36n_{11}+36n_{12}+36n_{13}+24n_{21}+24n_{22}+24n_{23}+12n_{31}+12n_{32}+12n_{33})q^{3}\right] \\ +p^{2}\left[6n_{11}+3n_{12}+6n_{21}+3n_{22}+6n_{31}+3n_{32}-\right. \\ (60n_{11}+42n_{12}+24n_{13}+54n_{21}+36n_{22}+18n_{23}+48n_{31}+30n_{32}+12n_{33})q+ \\ (126n_{11}+108n_{12}+90n_{13}+108n_{21}+90n_{22}+72n_{23}+90n_{31}+72n_{32}+54n_{33})q^{2}- \\ \left.(72n_{11}+72n_{12}+72n_{13}+60n_{21}+60n_{22}+60n_{23}+48n_{31}+48n_{32}+48n_{33})q^{3}\right]- \\ p^{3}\left[4n_{11}+2n_{12}+4n_{21}+2n_{22}+4n_{31}+2n_{32}-\right. \\ (36n_{11}+24n_{12}+12n_{13}+36n_{21}+24n_{22}+12n_{23}+36n_{31}+24n_{32}+12n_{33})q+ \\ (72n_{11}+60n_{12}+48n_{13}+72n_{21}+60n_{22}+48n_{23}+72n_{31}+60n_{32}+48n_{33})q^{2}- \\ \left.(40n_{11}+40n_{12}+40n_{13}+40n_{21}+40n_{22}+40n_{23}+40n_{31}+40n_{32}+40n_{33})q^{3}\right] \end{matrix}$$

$$\begin{matrix} a_{1}=p\left[(2n_{11}+n_{12}+n_{21}+n_{22})q+(-12n_{11}-9n_{12}-6n_{13}-6n_{21}-5n_{22}-3n_{23})q^{2}+\right. \\ \text{ }(18n_{11}+16n_{12}+14n_{13}+9n_{21}+8n_{22}+7n_{23})q^{3}+(-8n_{11}-8n_{12}-8n_{13}-4n_{21}- \\ \left.\text{ }4n_{22}-4n_{23})q^{4}\right]-p^{2}\left[(12n_{11}+6n_{12}+9n_{21}+5n_{22}+6n_{31}+3n_{32})q-\right. \\ \text{ }(60n_{11}+42n_{12}+24n_{13}+42n_{21}+29n_{22}+15n_{23}+24n_{31}+15n_{32}+6n_{33})q^{2}+ \\ \text{ }(84n_{11}+72n_{12}+60n_{13}+57n_{21}+48n_{22}+39n_{23}+30n_{31}+24n_{32}+18n_{33})q^{3}- \\ \text{ }\left.(36n_{11}+36n_{12}+36n_{13}+24n_{21}+24n_{22}+24n_{23}+12n_{31}+12n_{32}+12n_{33})q^{4}\right]+ \\ \text{ }p^{3}\left[(18n_{11}+9n_{12}+16n_{21}+8n_{22}+14n_{31}+7n_{32})q-\right. \\ \text{ }(84n_{11}+57n_{12}+30n_{13}+72n_{21}+48n_{22}+24n_{23}+60n_{31}+39n_{32}+18n_{33})q^{2}+ \\ \text{ }(114n_{11}+96n_{12}+78n_{13}+96n_{21}+80n_{22}+64n_{23}+78n_{31}+64n_{32}+50n_{33})q^{3}- \\ \text{ }\left.(48n_{11}+48n_{12}+48n_{13}+40n_{21}+40n_{22}+40n_{23}+32n_{31}+32n_{32}+32n_{33})q^{4}\right]- \\ \text{ }p^{4}\left[(8n_{11}+4n_{12}+8n_{21}+4n_{22}+8n_{31}+4n_{32})q-\right. \\ \text{ }(36n_{11}+24n_{12}+12n_{13}+36n_{21}+24n_{22}+12n_{23}+36n_{31}+24n_{32}+12n_{33})q^{2}+ \\ \text{ }(48n_{11}+40n_{12}+32n_{13}+48n_{21}+40n_{22}+32n_{23}+48n_{31}+40n_{32}+32n_{33})q^{3}- \\ \text{ }\left.(20n_{11}+20n_{12}+20n_{13}+20n_{21}+20n_{22}+20n_{23}+20n_{31}+20n_{32}+20n_{33})q^{4}\right] \end{matrix}$$

$$\begin{matrix} a_{0}=(1-p)^{2}p^{2}(1-q)^{2}q^{2}\left[2n_{21}+n_{22}-4n_{21}p-2n_{22}p-4n_{31}p-2n_{32}p+\right. \\ \text{ }4n_{11}(1-p)(1-q)-4n_{13}q-2n_{21}q-2n_{22}q-2n_{23}q+ \\ \text{ }\left.4n_{13}pq+4n_{21}pq+4n_{22}pq+4n_{23}pq+4n_{31}pq+4n_{32}pq+4n_{33}pq+2n_{12}(1-p)(1-2q)\right] \end{matrix}$$
